# Mental health attitudes in Malta: a cross-sectional survey exploring the knowledge and perceptions of general practitioner trainees

**DOI:** 10.1192/bjb.2023.56

**Published:** 2024-06

**Authors:** Daniela Zammit, Jonathan Grech, Patrick Abela, David Mamo

**Affiliations:** 1 Mount Carmel Hospital, Attard, Malta; 2 Malta College of Family Doctors, Gzira, Malta

**Keywords:** Mental health, general practitioners, opinions and attitudes, knowledge, stigma

## Abstract

**Aims and method:**

This study aimed to assess current levels of knowledge, opinions and attitudes regarding mental health among the local cohort of general practitioner trainees (*n* = 45) working in Malta. A questionnaire adapted from the Mental Health Literacy Scale was used. Data were analysed using one-way analysis of variance and Pearson correlation tests.

**Results:**

All participants had scores equal to or more than the mean score in their knowledge and confidence assessments; 51% of the participants achieved the maximum score for a very positive attitude towards mental health, with such scores found particularly among female trainees. Increased levels of knowledge are associated with a more positive attitude, which can in turn lead to greater acceptance and reduce stigma.

**Clinical implications:**

Knowledge is a powerful tool for reducing stigma and improving the doctor–patient relationship, indicating that regular training initiatives are necessary to equip budding general practitioner specialists with the necessary skills and confidence.

Primary healthcare focuses on addressing a person's health needs throughout their lifetime by adopting a biopsychosocial approach.^1^ It incorporates health promotion and patient education; prevention, early identification and treatment of diseases; regular follow-up and rehabilitation; and even palliative care towards the end of life.

Modern mental health services are shifting away from traditional hospital-based care and moving towards community-based services.^2^ In 2001, the World Health Organization (WHO) highlighted the vital need to integrate mental healthcare into the primary healthcare system.^3^ Later, the WHO European Action Plan (2013)^4^ emphasised that this approach will not only help to identify and treat acute mental illness effectively but also provide an opportunity for individuals and their families to rehabilitate back into the community and improve their quality of life. However, this does not come without its challenges, namely the barriers of stigma, attitudes, lack of confidence in the clinician's skills and access to care.

Some studies have found that more experienced general practitioners (GPs) may have more stigmatising attitudes towards mental illness compared with their younger successors.^5^ The present study focuses on junior GP trainees who are still at the start of their careers and uses a cross-sectional survey to gain a better understanding of the opinions and attitudes of local GP trainees as budding specialists. Our aim was to assess current levels of knowledge, opinions and attitudes regarding mental health among GP trainees working in Malta, to help inform future educational initiatives.

## Method

An anonymous questionnaire was distributed among the local cohort of GP trainees using questions from the validated Mental Health Literacy Scale^6^ (MHLS) to assess their knowledge and opinions on various aspects of mental health (answers were scored using Likert scales). This tool has been widely used to assess variations in mental health literacy (MHL), comparing different populations as well as individuals before and after an intervention. We also gathered basic demographic information about each participant, providing us with their age, gender, years in GP training and whether they had ever received formal training in mental healthcare (Table 1). All GP trainees had completed their undergraduate medical training in Malta.

**Table 1:** Demographic variables for participants

Measure		Sample size	Percentage (%)
Gender	Male	12	26.7
	Female	33	73.3
Age, years	23–25	14	31.1
	26–28	22	48.9
	29–31	9	20.0
Years in general practice training	1	20	44.4
	2	13	28.9
	3	12	26.7
Received prior formal psychiatric training	Yes	12	26.7
	No	33	73.3

The MHLS is a 35-item questionnaire containing questions related to the subthemes of general knowledge (15 items), confidence in accessing mental health information (four items), opinions and attitudes (nine items) and acceptance (seven items).

Questions from the knowledge subtheme test the individual's ability to recognise mental disorders by presenting small clinical vignettes and asking them to rate the likelihood of a particular mental health disorder (as stated in the ICD-10 classification system), along with knowledge on risk factors and causes of mental illness, self-treatment and patient confidentiality. The knowledge score is generated by averaging the ratings provided for the 15 items, measured on a four-point Likert scale. Knowledge scores range from 0 to 3, where 0 corresponds to very poor knowledge and 3 corresponds to very good knowledge.

The four questions pertaining to the confidence subtheme ask participants about how confident they are in knowing how to access or seek information about mental illness when in need. The confidence score is generated by averaging the ratings provided for the four items, measured on a five-point Likert scale. Confidence scores range from 0 to 4, where 0 corresponds to very low confidence and 4 corresponds to very high confidence.

The opinions and attitude subtheme centres on common mental health myths that would usually promote pessimistic views. Participants answer questions asking them whether they believe that a mental illness is a sign of personal weakness or a fake medical illness, or that people with a mental health diagnosis can ‘snap out of it’ if they want to and are dangerous and to be avoided. In addition, this section asks participants whether they would readily disclose that they suffer from a mental illness and/or seek help from professionals. This section uses reverse scoring, with scores generated by averaging the ratings provided for nine items, measured on a five-point Likert scale. Opinion and attitude scores range from 0 to 4, where 0 corresponds to a very negative attitude towards mental illness and 4 corresponds to a very positive attitude.

Finally, the acceptance subtheme presents the participants with real-life scenarios and asks questions about how willing they would be to accept, for instance, being neighbours with, socialising with, making friends with, marrying into the family of, working closely at work with or employing someone with mental illness, or voting for a politician with a mental illness. The acceptance score is generated by averaging the ratings provided for seven items, measured on a five-point Likert scale. Acceptance scores range from 0 to 4, where 0 corresponds to very poor acceptance (i.e. high stigma) and 4 corresponds to very high acceptance (i.e. low stigma).

This study was performed in accordance with the Declaration of Helsinki. Ethics approval was provided by the Data Protection Office of Mental Health Services, Malta, and the Postgraduate Training Committee of the Department of Primary Healthcare, Malta. All the GP trainees present after a weekly lecture were offered the opportunity to voluntarily participate in our study. Verbal consent was obtained from each adult participant, and completing and submitting the anonymous questionnaire was considered to constitute consent to participating in the study. No identifying details (including name, initials and employee number) were noted, and thus neither the participant nor their responses could be traced. Moreover, whether any specific trainee participated in the study or not could not be traced.

Data were analysed using SPSS version 25. One-way analysis of variance was used to test for any correlations of the various subthemes with demographic factors (age, gender, years in GP practice, and whether any formal psychiatric training had been received). In addition, Pearson correlation tests were used to explore relationships among the various subthemes.

## Results

A total of 45 completed questionnaires were received, resulting in an 82% response rate.

In the knowledge and confidence sections, all participants had scores equal to or greater than the median score, demonstrating a high ability to correctly diagnose a clinical scenario and access or seek information about a mental illness when necessary. In the opinions and attitudes section, 23 of the 45 participants achieved the highest score, corresponding to a very positive attitude towards mental health in general (Supplementary Material available at https://doi.org/10.1192/bjb.2023.56).

In the acceptance section, where low scores corresponded to very poor acceptance (i.e. high stigma) and high scores corresponded to very high acceptance (i.e. low stigma) of people with mental illness, 14 participants achieved the mean score, and another nine had below-average scores, indicating poor acceptance and high stigma. The remaining half of the participants (22 in total) had higher-than-average scores, meaning higher acceptance and low stigma.

### Demographic factors

Females had statistically significantly higher scores than males for opinions and attitudes (*P* = 0.022), meaning that they had more positive attitudes. However, there was no significant gender discrepancy for the other subthemes (knowledge, confidence and acceptance). Half of the participants (49%) were aged between 26 and 28 years. Age was not found to be a significant determinant of score, as there was no age discrepancy for any of the four subthemes tested, and nor was previous experience with formal psychiatric training.

We analysed whether the questionnaire's results could be affected by how long the participant had been working as a GP trainee. There was a significant difference for the knowledge subtheme, with first- and third-year GP trainees scoring higher than the second-year trainees (*P* < 0.001). In addition, for the opinions and attitudes section, first-year GP trainees had a significantly higher mean score than second- and third-year trainees, meaning that they had the most positive attitudes towards mental health of all three years of experience (*P* = 0.014). For the other subscales, there was no discrepancy among the groups clustered by years in GP practice.

### Correlation testing

**Fig. 1:**
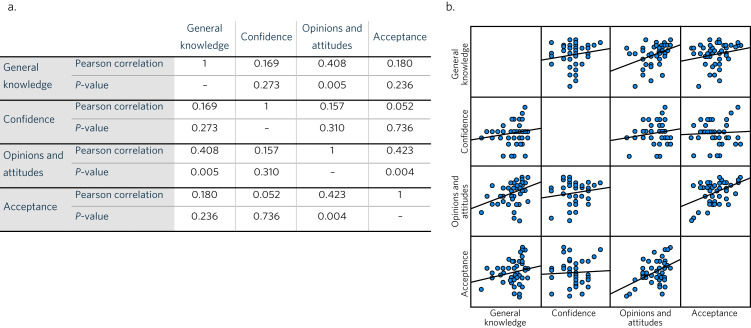
(a) Pearson correlation coefficients measuring the strengths of associations among all subthemes. (b) Pearson correlation scatterplot.

As shown in Fig. 1, knowledge had a statistically significant positive relationship with opinions and attitudes; therefore, a high score for the former would mean a similar positive score would be expected for attitudes towards mental health. The opinions and attitudes subtheme also showed a significantly positive relationship with both knowledge and acceptance, with *P*-values indicating a <0.05 level of significance. Despite this finding, having a high score for knowledge was not directly statistically associated with acceptance. Although all other pairwise relationships were found to be positive, the associations were weak (the *P*-values exceeded the 0.05 level of significance).

## Discussion

The present study is the first to look at knowledge, attitudes and perceptions towards mental health specifically among GP trainees in their early careers. It considers the relationships among these variables, together with other demographic factors that can influence clinical practice when managing individuals with mental illnesses in primary healthcare.

The MHLS was developed by Matt O'Connor as a validated scale-based measure of the various attributes of MHL.^6^ MHL refers to knowledge and attitudes in relation to mental health and consists of seven important characteristics as described by Jorm et al:^7^ the ability to recognise specific mental disorders; knowing how to seek information about mental health; knowing the risk factors and causes of mental disorders; knowledge and awareness of self-treatments; knowing what professional help is available; and attitudes that encourage recognition and appropriate help-seeking. As a result, having sound MHL promotes timely recognition, management and prevention of mental health issues.

Our results obtained for the questions pertaining to the knowledge subtheme showed that all participants had above-average scores for general knowledge, demonstrating a high ability to correctly diagnose a clinical scenario. There was a significant drop in knowledge scores between first-year and second-year GP trainees, but scores then increased again during the third year of training, as confirmed by Tukey *post hoc* testing. Our findings offer no obvious explanation for this observation. We conclude that the results are specific to this particular cohort of second-year GP trainees and cannot necessarily be generalised. Similarly, our results for questions concerning confidence demonstrated that all participants had encouraging above-average scores.

Overall, our results for the opinions and attitudes subtheme showed that the majority of the GP trainees had a positive attitude towards mental health, especially the female trainees, who scored significantly higher than their male counterparts, and those in their first year of their GP training. This is consistent with the findings of similar studies that have shown how certain demographic factors of professionals can affect the care of their patients.^8–10^ This subtheme highlights that a positive attitude promotes recognition and appropriate help-seeking behaviours when required.

### Comparisons with findings from other studies

Similar to our study, several others have looked at possible factors that might influence a doctor's attitude to treating mental disorders, to help identify what can be improved, provide better care to patients and reduce the stigma within our profession.

Our study participants were mostly young adult trainees at the start of their careers, with ages ranging between 23 and 31 years. In our study sample, age was not a significant factor affecting our results. However, a recent systematic review highlighted that older and more experienced doctors in the primary care setting tend to exhibit more stigmatising attitudes towards individuals with mental illness.^5^ Vistorte et al argued that older doctors tend to show more stigma and prejudice towards their patients in comparison with their younger and less-experienced counterparts, partly as a result of a lack of consistent training.

Different clinical diagnoses may evoke different reactions from doctors, and these may vary among specialties.^11^ Individuals that misuse illegal substances and those with self-harming behaviour tend to generate more stigma and negative attitudes from healthcare professionals,^12^ whereas those individuals who attempt suicide receive more empathy. Psychiatrists and GPs have been shown to have more positive attitudes towards individuals who are suicidal compared with doctors working in internal medicine, particularly among female physicians, as corroborated by our results. Similarly, patients diagnosed with schizophrenia receive more stigmatising attitudes from primary care physicians in comparison with patients with depression and are less likely to be treated adequately in the primary healthcare system.^13–15^ GPs differ from psychiatrists in terms of their attitudes to treating depression, which will in turn affect their management plans.^16–18^

In our study, Pearson correlation testing confirmed the presence of positive, strong correlations of knowledge with opinions and attitude and with acceptance. Increased levels of knowledge are associated with adopting more positive attitudes towards mental health, which can in turn lead to higher acceptance and reduced stigma. Although correlation testing did not show knowledge to have a direct relationship with acceptance, it is still considered to be a prerequisite for continuing elimination of stigma and prejudice.

In fact, there is accumulating evidence that knowledge is the key to breaking barriers and stigma, leading to several training programmes being developed to help promote positive attitudes towards mental health. OSPI-EUROPE (Optimizing suicide prevention programs and their implementation in Europe) is an evidence-based multi-level approach with the goal of optimising suicide-prevention interventions implemented in Europe.^19^ One of the five-level approaches included training sessions and videos aimed at primary care physicians and was found to be effective at improving the skills of GPs, as well as their attitudes and confidence in managing depression and suicide prevention.^20^

In 2006, Üçok et al compared the views and attitudes of GPs before and after a brief anti-stigma educational session with regard to schizophrenia. Three months after this educational intervention, the GPs’ attitudes showed a statistically significant positive change, suggesting that brief training sessions (as well as other interventions) can be helpful in improving approaches towards severe mental illnesses.^9^ Although stigma remains a complex phenomenon, educational programmes are an essential step in breaking the taboo.

A doctor's positive attitude is central to encouraging patients to take responsibility for their own mental health self-care. On the other hand, misinformation, misconceptions and lack of supportive training to doctors all contribute to negative attitudes and can in turn influence the healthcare professional's decision-making in their daily practice.^21^ Patients can pick up such negative perceptions, which will affect their willingness to engage with mental health services and adhere to their management plans.^22^ Evidence shows that GPs that work closely with community mental health teams have better perceptions of mental health treatment and less stigmatising attitudes.^23^ Improving collaborations between specialist mental health services and primary healthcare can be an asset in delivering good-quality care to patients.

### Strengths and limitations

The main strength of our study is that, to our knowledge, it is the first study to specifically assess knowledge, perceptions and attitudes regarding mental health among all GP trainees specialising in Malta at the start of their careers. The main limitation is that we did not ask for further background information from each trainee, such as whether they had had first-hand experience with mental health struggles or whether they had undergraduate exposure to mental-health-based teaching and/or clinical placements. These factors could have influenced their responses to questions about opinions and attitudes and perhaps even acceptance, and considering such factors could have enabled us to determine the influence of the level and/or duration of undergraduate exposure on MHLS scores.

Nevertheless, our study provides further proof that encouraging educational initiatives for GPs can make them feel more confident and tolerant when working with individuals with mental health issues. We suggest that similar studies are carried out after any training initiative that is rolled out to assess for any improvements in levels of knowledge, attitudes and confidence. Moreover, additional studies to look for any differences in knowledge and attitudes between GP trainees and more experienced GPs could help to shed light on any differences in stigmatising behaviour between these groups.

### Clinical implications

Mental health is becoming a top priority in the nation's agenda, with a push towards community mental health treatment and a shift in focus to the primary healthcare system as the front line. We need to continue working on breaking barriers by addressing the stigma surrounding mental illnesses and promoting positive attitudes. Our study shows that knowledge can be a powerful tool in achieving this, and regular training initiatives can equip budding GPs with much needed confidence.

## Supporting information

Zammit et al. supplementary material Zammit et al. supplementary material

## Data Availability

The data supporting the findings of this study are available within the article and/or its supplementary material.

## About the authors

**Daniela Zammit**, M.D. (Melit), MRCPsych, Specialist Trainee in Psychiatry, Psychiatry, Mount Carmel Hospital, Attard, Malta; **Jonathan Grech**, M.D. (Melit), Specialist Trainee in General Practice, Department of Family Medicine, Malta College of Family Doctors, Gzira, Malta; **Patrick Abela**, M.D. (Melit), CCT Psych, Psychiatrist, Psychiatry, Mount Carmel Hospital, Attard, Malta; **David Mamo**, M.D. (Melit), M.Sc. (Lond.), M.S.(Tor.), F.R.C.P.C., F.A.P.A., Dip. A.B.P.N, Consultant Psychiatrist and Visiting Associate Professor, Psychiatry, Mount Carmel Hospital, Attard, Malta.
