# Supplementary material for: Mental health attitudes in Malta: a cross-sectional survey exploring the knowledge and perceptions of general practitioner trainees
Source: BJPsych Bull. 2024 Jun;48(3):168–72. doi: 10.1192/bjb.2023.56 (PMC11134023; doi:10.1192/bjb.2023.56)
Supplement: Zammit et al. supplementary material [file S2056469423000566sup001.docx]

**Annexes (Supplementary material)**


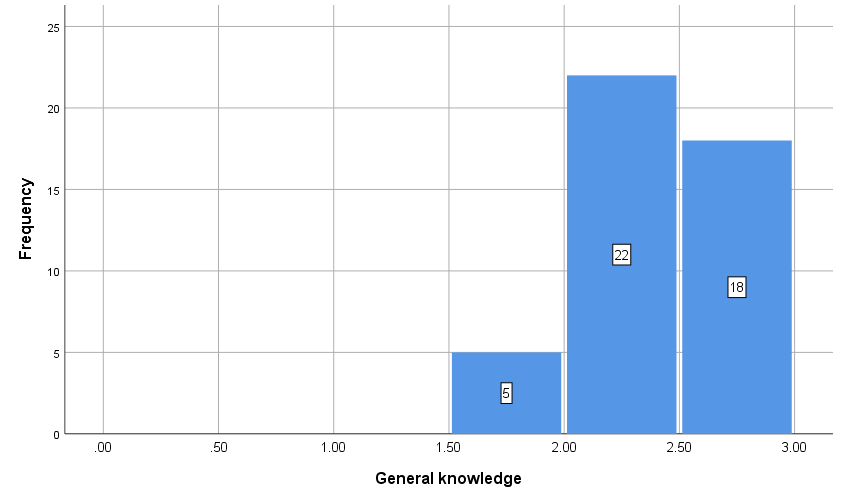


**Figure 1: General knowledge (x-axis) vs. frequency of participants (y-axis).** The mean “Knowledge” score on the Mental Health Literacy Scale (MHLS) ranges from 0 to 3, where 0 corresponds to very poor knowledge and 3 corresponds to very good knowledge.


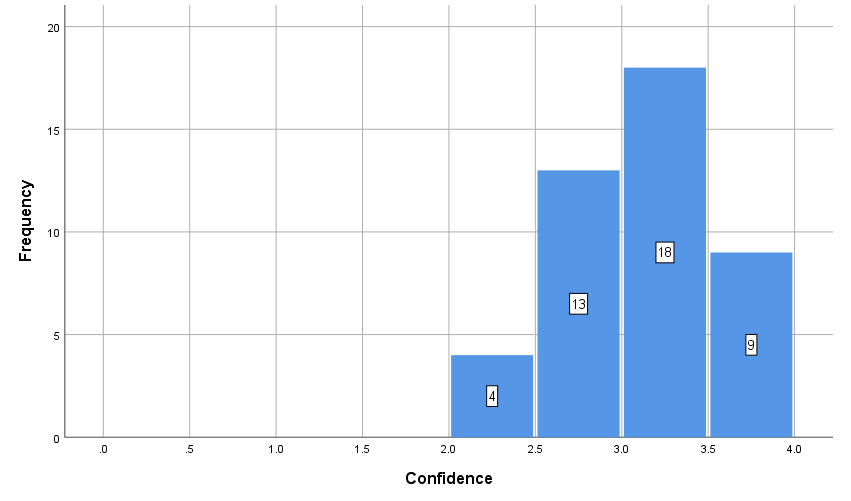


**Figure 2: Confidence of seeking information (x-axis) vs. frequency of participants (y-axis).** The mean “Confidence” score on the Mental Health Literacy Scale (MHLS) ranges 0 to 4, where 0 corresponds to very low confidence and 4 corresponds to very high confidence.


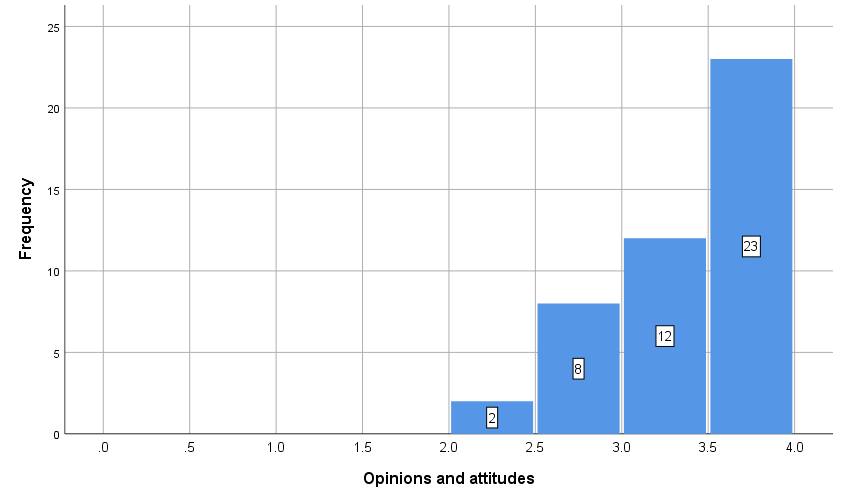


**Figure 3: Opinions and Attitude (x-axis) vs. frequency of participants (y-axis).** The mean “Opinion & Attitude” score on the Mental Health Literacy Scale (MHLS) ranges from 0 to 4, where 0 corresponds to a very negative attitude towards mental illness and 4 corresponds to a very positive attitude.


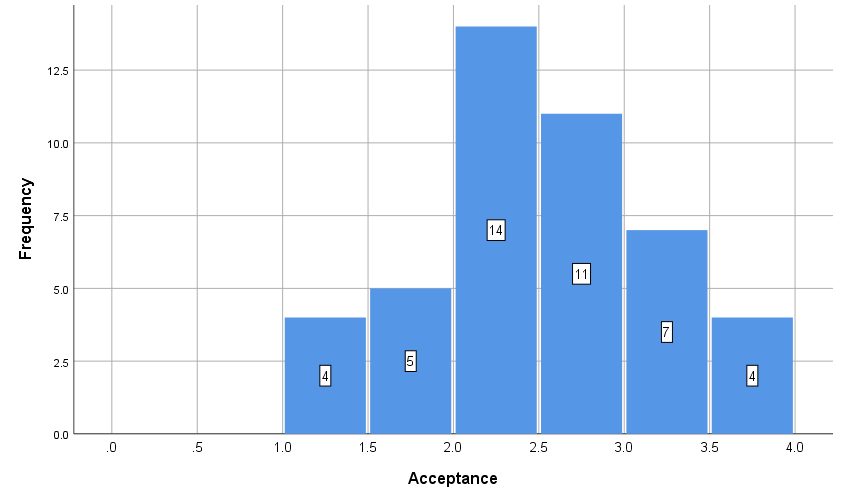


**Figure 4: Acceptance (x-axis) vs. frequency of participants (y-axis).** The mean “Acceptance” score on the Mental Health Literacy Scale (MHLS) ranges from 0 to 4, where 0 corresponds to very poor acceptance (i.e. high stigma) and 4 corresponds to very high acceptance (i.e. low stigma).

|  | | Sample Size | Mean | Std. Deviation | P-value |
| --- | --- | --- | --- | --- | --- |
| General knowledge | Male | 12 | 2.40 | 0.316 | 0.654 |
|  | Female | 33 | 2.37 | 0.265 |  |
| Confidence | Male | 12 | 2.92 | 0.404 | 0.778 |
|  | Female | 32 | 2.96 | 0.480 |  |
| Opinions and attitudes | Male | 12 | 3.11 | 0.446 | **0.022** |
|  | Female | 33 | 3.47 | 0.417 |  |
| Acceptance | Male | 12 | 2.35 | 0.747 | 0.440 |
|  | Female | 33 | 2.53 | 0.723 |  |

**Table 1: One-way ANOVA: Gender differences in the Mental Health Literacy Scale (MHLS) sub-themes.**

|  | | Sample size | Mean | Std. Deviation | P-value |
| --- | --- | --- | --- | --- | --- |
| General knowledge | 23-25 | 14 | 2.35 | 0.266 | 0.521 |
|  | 26-28 | 22 | 2.34 | 0.317 |  |
|  | 29-31 | 9 | 2.46 | 0.170 |  |
| Confidence | 23-25 | 14 | 2.91 | 0.411 | 0.836 |
|  | 26-28 | 21 | 2.94 | 0.558 |  |
|  | 29-31 | 9 | 3.03 | 0.232 |  |
| Opinions and attitudes | 23-25 | 14 | 3.47 | 0.380 | 0.537 |
|  | 26-28 | 22 | 3.30 | 0.457 |  |
|  | 29-31 | 9 | 3.33 | 0.530 |  |
| Acceptance | 23-25 | 14 | 2.22 | 0.613 | 0.122 |
|  | 26-28 | 22 | 2.71 | 0.706 |  |
|  | 29-31 | 9 | 2.35 | 0.839 |  |

**Table 2: One-way ANOVA: Age-group differences in the Mental Health Literacy Scale (MHLS) sub-themes.**

|  | | Sample size | Mean | Std. Deviation | P-value |
| --- | --- | --- | --- | --- | --- |
| General knowledge | 1 year | 20 | 2.46 | 0.194 | **0.000** |
|  | 2 years | 13 | 2.10 | 0.322 |  |
|  | 3 years | 12 | 2.49 | 0.108 |  |
| Confidence | 1 year | 20 | 2.88 | 0.401 | 0.218 |
|  | 2 years | 12 | 2.88 | 0.406 |  |
|  | 3 years | 12 | 3.15 | 0.558 |  |
| Opinions and attitudes | 1 year | 20 | 3.56 | 0.295 | **0.014** |
|  | 2 years | 13 | 3.12 | 0.339 |  |
|  | 3 years | 12 | 3.28 | 0.611 |  |
| Acceptance | 1 year | 20 | 2.44 | 0.602 | 0.875 |
|  | 2 years | 13 | 2.57 | 0.625 |  |
|  | 3 years | 12 | 2.48 | 1.022 |  |

**Table 3: One-way ANOVA: Years in General Practitioner (GP) practice differences in the Mental Health Literacy Scale (MHLS) sub-themes**

**Appendix:**

**Mental Health Literacy Scale**

*The purpose of these questions is to gain an understanding of your knowledge of various aspects to do with mental health. When responding, we are interested in your degree of knowledge.*

*Kindly circle the most likely correct answer.*

**1.** If someone became extremely nervous or anxious in one or more situations with other people (e.g., a party) or performance situations (e.g., presenting at a meeting) in which they were afraid of being evaluated by others and that they would act in a way that was humiliating or feel embarrassed, then to what extent do you think it is likely they have **Social Phobia**.

*Very unlikely Unlikely Likely Very Likely*

**2.** If someone experienced excessive worry about a number of events or activities where this level of concern was not warranted, had difficulty controlling this worry and had physical symptoms such as having tense muscles and feeling fatigued then to what extent do you think it is likely they have **Generalised Anxiety Disorder**.

*Very unlikely Unlikely Likely Very Likely*

**3.** If someone experienced a low mood for two or more weeks, had a loss of pleasure or interest in their normal activities and experienced changes in their appetite and sleep then to what extent do you think it is likely they have **Major Depressive Disorder.**

*Very unlikely Unlikely Likely Very Likely*

**4.** To what extent do you think it is likely that **Personality Disorders** are a category of mental illness:

*Very unlikely Unlikely Likely Very Likely*

**5.** To what extent do you think it is likely that **Dysthymia** is a disorder:

*Very unlikely Unlikely Likely Very Likely*

**6.** To what extent do you think it is likely that the diagnosis of **Agoraphobia** includes **anxiety** about situations where escape may be difficult or embarrassing?

*Very unlikely Unlikely Likely Very Likely*

**7.** To what extent do you think it is likely that the diagnosis of **Bipolar Disorder** includes experiencing periods of elevated (i.e., high) and periods of depressed (i.e., low) mood?

*Very unlikely Unlikely Likely Very Likely*

**8.** To what extent do you think it is likely that the diagnosis of **Drug Dependence** includes physical and psychological tolerance of the drug (i.e., require more of the drug to get the same effect)?

*Very unlikely Unlikely Likely Very Likely*

**9.** To what extent do you think it is likely that in general, in Malta, women are **more** likely to experience a **mental illness of any kind** compared to men?

*Very unlikely Unlikely Likely Very Likely*

**10.** To what extent do you think it is likely that in general, in Malta, men are **more** likely to experience an **anxiety disorder** compared to women?

*Very unlikely Unlikely Likely Very Likely*

**11.** To what extent do you think it would be helpful for someone to improve their **quality of sleep** if they were having difficulties managing their emotions (e.g., becoming very anxious or depressed)?

*Very unhelpful Unhelpful Helpful Very helpful*

**12.** To what extent do you think it would be helpful for someone to **avoid all activities or situations** that made them feel **anxious** if they were having difficulties managing their emotions?

*Very unhelpful Unhelpful Helpful Very helpful*

**13.** To what extent do you think it is likely that **Cognitive Behaviour Therapy (CBT)** is a therapy based on challenging negative thoughts and increasing helpful behaviours?

*Very unlikely Unlikely Likely Very Likely*

**14.** Mental health professionals are bound by **confidentiality**; however there are certain conditions under which this does not apply. To what extent do you think it is likely that the following is a condition that would allow a mental health professional to break confidentiality?

i. If you are at **immediate risk** of harm to yourself or others

*Very unlikely Unlikely Likely Very Likely*

ii. if your problem is **not life-threatening** and they want to assist others to better support you

*Very unlikely Unlikely Likely Very Likely*

**16.** I am confident that I know where to **seek information** about mental illness:

*Strongly Disagree Disagree Neither agree or disagree Agree Strongly agree*

**17.** I am confident using the computer or telephone to seek information about mental illness:

*Strongly Disagree Disagree Neither agree or disagree Agree Strongly agree*

**18.** I am confident attending **face to face appointments** to seek information about mental illness

(e.g., seeing the GP):

*Strongly Disagree Disagree Neither agree or disagree Agree Strongly agree*

**19.** I am confident I have **access to resources** (e.g., GP, internet, friends) that I can use to seek information about mental illness:

*Strongly Disagree Disagree Neither agree or disagree Agree Strongly agree*

**20.** People with a mental illness could **snap out** if it if they wanted:

*Strongly Disagree Disagree Neither agree or disagree Agree Strongly agree*

**21.** A mental illness is a sign of **personal weakness:**

*Strongly Disagree Disagree Neither agree or disagree Agree Strongly agree*

**22.** A mental illness is **not a real medical illness:**

*Strongly Disagree Disagree Neither agree or disagree Agree Strongly agree*

**23.** People with a mental illness are **dangerous:**

*Strongly Disagree Disagree Neither agree or disagree Agree Strongly agree*

**24.** It is best to **avoid people with a mental illness** so that you don't develop this problem:

*Strongly Disagree Disagree Neither agree or disagree Agree Strongly agree*

**25.** If I had a mental illness, I **would not tell anyone:**

*Strongly Disagree Disagree Neither agree or disagree Agree Strongly agree*

**26.** Seeing a mental health professional means **you are not strong enough** to manage your own difficulties:

*Strongly Disagree Disagree Neither agree or disagree Agree Strongly agree*

**27.** If I had a mental illness, I **would not seek help** from a mental health professional:

*Strongly Disagree Disagree Neither agree or disagree Agree Strongly agree*

**28.** I believe treatment for a mental illness, provided by a **mental health professional**, would not be effective:

*Strongly Disagree Disagree Neither agree or disagree Agree Strongly agree*

**29.** How willing would you be to **move next door** to someone with a mental illness?

*Definitely unwilling ; Probably unwilling ; Neither unwilling or willing ; Probably willing ; Definitely willing*

**30.** How willing would you be to **spend an evening socialising** with someone with a mental illness?

*Definitely unwilling ; Probably unwilling ; Neither unwilling or willing ; Probably willing ; Definitely willing*

**31.** How willing would you be to **make friends** with someone with a mental illness?

*Definitely unwilling ; Probably unwilling ; Neither unwilling or willing ; Probably willing ; Definitely willing*

**32.** How willing would you be to have someone with a mental illness start **working closely** with you on a job?

*Definitely unwilling ; Probably unwilling ; Neither unwilling or willing ; Probably willing ; Definitely willing*

**33.** How willing would you be to have someone with a **mental illness marry into your family**?

*Definitely unwilling ; Probably unwilling ; Neither unwilling or willing ; Probably willing ; Definitely willing*

**34.** How willing would you be to **vote for a politician** if you knew they had suffered a mental illness?

*Definitely unwilling ; Probably unwilling ; Neither unwilling or willing ; Probably willing ; Definitely willing*

**35.** How willing would you be to **employ someone** if you knew they had a mental illness?

*Definitely unwilling ; Probably unwilling ; Neither unwilling or willing Probably willing ; Definitely willing*

**Reference**: O’Connor, M., & Casey, L. (2015). The mental health literacy scale (MHLS): A new scale-based measure of mental health literacy, Psychiatry Research.

*The MHLS was used with the kind permission of Dr. Matt O’Connor*
